# Intersectional HIV stigma in Sub-Saharan Africa, Latin America and the Caribbean: Insights and pathways forward – A scoping review

**DOI:** 10.1371/journal.pgph.0004240

**Published:** 2025-02-10

**Authors:** Nipher Malika, Laura M. Bogart, Joseph K. B. Matovu, Nthabiseng Phaladze, Kuraish Mubiru, Maria Leon Rhandomy, Yeycy Donastorg, Javier Valencia Huamani, Onalethata Mpebe, Nixon Chisonga, Emelda Fonki, Lejeune Y. Lockett, David Lee

**Affiliations:** 1 RAND, Santa Monica, California, United States of America; 2 Charles R. Drew University of Medicine and Sciences, Los Angeles, California, United States of America; 3 Makerere University School of Public Health, Kampala, Uganda; 4 Busitema University Faculty of Health Sciences, Mbale, Uganda; 5 University of Botswana, School of Nursing, Gaborone, Botswana; 6 Uganda Young Positives, Kampala, Uganda; 7 Peru Clinical Trials Unit, Lima, Peru; 8 Instituto Dermatologico y Cirugia de la Piel, Santo Domingo, Dominican Republic; 9 Asociacion Civil Impacta Salud y Educacion, Lima, Peru; 10 Positive Moments Organization, Mmopane, Botswana; 11 Mulungushi University, Kabwe, Zambia; 12 APLA Health, Los Angeles, California, United States of America; PLOS: Public Library of Science, UNITED STATES OF AMERICA

## Abstract

Research has recently surged on intersectional HIV stigma, including how intersecting stigmatized identities and socio-structural conditions influence HIV prevention and treatment outcomes. However, most of this work has been concentrated in high-income settings. This scoping review aimed to provide an overview of research on intersectional HIV stigma in Sub-Saharan Africa, Latin America and the Caribbean. A search was conducted using five databases for articles published between January 2008 and April 2023. Two reviewers independently screened all identified studies, sorted the included studies, and conducted descriptive analyses. Of 1907 retrieved studies, 73 met inclusion criteria, of which 16% were intervention studies and 84% were non-intervention studies. Stigma was propagated through structural factors (e.g., anti-sexual and gender minority laws), institutional factors, and socio-demographic factors. Moreover, place-based differences emerged. Findings of the scoping review were discussed and interpreted by a community advisory board composed of activists and researchers from Sub-Saharan Africa, Latin America, and the Caribbean, which provided recommendations on the pathways forward in research for intersectional HIV stigma. Future research on intersectional stigma should encompass social marketing studies for promoting inclusive HIV services, strategies to transform the narrative in media, and investigations into the impact of laws against sexual and gender minority (SGM) individuals on HIV service participation, all approached from the perspective of those affected by the intersectional stigma.

## Introduction

Since the early days of the HIV epidemic, misconceptions have fueled fear and prejudice, leading to stigma against those affected [1]. Acknowledging how power dynamics intersect to shape social interactions, researchers have embraced an intersectional approach to studying how HIV stigma intertwines with other prejudices such as racism, ageism, classism, ableism, and cis-genderism, amplifying the epidemic’s impact on affected communities [2–9]. However, most intersectional HIV stigma studies to date have been conducted in high-income settings like the United States, concentrating on the interplay between race and sexual and gender identity [6,10–19].

HIV stigma intersectionalities identified in research in higher-income countries might not hold the same significance in low- and middle-income countries that are highly affected by HIV. For example, less is known about the landscape of intersectional stigma in regions of the world with generalized epidemics, where HIV prevalence is relatively high overall as well as in marginalized and vulnerable populations (i.e., “key populations,” as designated by PEPFAR and UNAIDS), which includes sex workers, transgender individuals, people who inject drugs, incarcerated people, and men who have sex with men [20,21].

The purpose of this study was twofold: 1) to review research on HIV associated intersectional stigmas and their impact on HIV care and treatment outcomes in Latin America, the Caribbean, and Sub-Saharan Africa through a scoping review of the literature, and 2) to engage with community and academic experts to determine next steps in HIV stigma research based on their in-country experiences and interpretation of prior research results. We sought to better understand the progress of and gaps in research in this space, distinguishing characteristics of studies in each region.

## Methods

### Global community advisory board (CAB) engagement

This scoping review was conducted in partnership with The University of California, Los Angeles-Charles R. Drew University of Medicine and Science (UCLA-CDU) Center for AIDS Research (CFAR) Global Community Advisory Board (CAB). CABs are an integral part of ensuring community involvement in HIV research [22,23]. CABs provide important feedback to researchers and link academic experts with community experts to ensure that research is relevant, acceptable, and ethical. CABs are invaluable for setting research priorities of relevance to communities, providing input on recruiting, and retaining participants, and educating the community about HIV research, treatment, and prevention [24–26].

The UCLA-CDU CFAR was established in 2022 with the mission of stopping HIV in Los Angeles County and beyond through engaging diverse researchers with multidisciplinary perspectives. The UCLA-CDU CFAR includes a Community Engagement and Clinical Informatics Core, which houses two CABs, a local, Los Angeles County CAB and a Global CAB. The Global CAB provides community input into HIV research and links local and international investigators to community advocates internationally; one of the main focuses of the Global CAB has been to provide an international perspective on intersectional stigma, and the Global CABs activities in this area provided an impetus for the present paper. Their members aimed to delve deeper into the landscape of intersectional stigma research within their regions and advocate for increased research efforts to address intersectional stigma within their respective countries and regions globally.

The Global CAB is led by three faculty co-chairs that represent global and community faculty researchers at CDU (a dually designated Historically Black Graduate Institution and Hispanic Serving Health Professions School). The CAB’s co-chairs’ primary role is to coordinate meetings, suggest meeting topics, and facilitate discussion. The co-chairs do not unilaterally shape the meeting agenda; instead, they provide non-directive support to enable meaningful participation from all CAB members. The Global CAB is comprised of academic and clinical researchers, community activists, advocates and people with lived experiences. Eleven Global CAB members representing six countries in Latin America the Caribbean (Brazil, Dominican Republic, Peru), and Sub-Saharan Africa (Botswana, Uganda, Zambia) contributed to discussions about intersectional stigma. The meetings took place once a month for one hour over a span of 15 months. CAB member recruitment was done through network contacts of CDU faculty members. CAB meetings were held in English. A $75 stipend was provided for attendance at each monthly one-hour meeting. The CAB ground rules specify that all members participate, and sufficient time is provided within each meeting for discussion, which ensured that a wide range of perspectives on intersectional stigma, as well as actionable recommendations for addressing stigma, were captured. Members from each country are individually encouraged to contribute their unique insights and experiences. The aim is to foster an environment where global perspectives are not only included but are central to the research.

### Protocol

A scoping review protocol, available from the corresponding author upon request, was compiled using guidance from Arksey and O’Malley [27].

### Data sources and search strategy

Guidelines [27–29] for scoping reviews were followed and results are reported in accordance with the Preferred Reporting Items for Systematic Reviews and Meta-Analyses extension for scoping reviews (PRISMA-ScR; see S1Text) [30]. The search was conducted between April and May of 2023 using PubMed, PsycINFO (EBSCOhost), Social Sciences Abstracts (EBSCOhost), Social Sciences Citation Index (ProQuest) and Google Scholar. Papers published between January 2008 and April 2023 were included. We restricted our search to papers published within the past 15 years because there was a scarcity of studies addressing HIV intersectional stigma prior to that timeframe. Articles were restricted to those published in English and those that discussed at least one or more types of stigma related to HIV risk factors, prevention, intervention, or care. We chose to focus on English-published studies due to several key factors. Resource limitations made translating articles from multiple languages impractical, and our reviewers’ proficiency in English ensured accurate interpretation. Focusing on English articles also provided consistency in evaluation, as all reviewers could uniformly apply inclusion criteria and quality assessment tools. Additionally, many major academic databases and high-impact journals predominantly publish in English, making these sources more accessible and increasing the likelihood of accessing high-quality studies. This approach allowed us to manage the scope of our review effectively while ensuring comprehensive and reliable findings.

Our inclusion criteria was influenced by the research aim and structured by PICO/PECO (Population, Intervention, Comparison, Outcome/Population Exposure, Comparison, Outcome) framework [31–33]. We sought to use these frameworks as opposed to others (e.g., SPIDER) due to literature demonstrating that PICO and PECO offer equal or higher sensitivity in identifying relevant studies [34]. This is particularly important for our research objectives, as these frameworks are well-suited for structuring clinical and epidemiological research questions. By employing PICO and PECO, we aimed to ensure a comprehensive and systematic approach to our study design and literature review, thereby enhancing the robustness and reliability of our findings.

We first identified our population of interest (e.g., individuals with HIV, AIDS, or HIV/AIDS), the concept (e.g., stigma, intersectional stigma, or related terms like intersectional discrimination), and the context (e.g., healthcare, community). We also conducted preliminary searches to understand the breadth and depth of the existing literature. This involved performing exploratory searches and identifying relevant keywords, synonyms, and Medical Subject Headings (MeSH) terms. To enhance our search strategy, we utilized truncation and Boolean operators [35,36]. For example, we used the search terms: 1. `HIV OR AIDS OR HIV/AIDS` to capture various expressions related to the human immunodeficiency virus and acquired immunodeficiency syndrome. 2. `stigmat* OR “intersectional stigma” OR “intersectional discrimination”` to include different forms and synonyms of stigma, such as “stigma,” “stigmatize,” and “stigmatization.” By employing truncation (e.g., `stigmat*`) and Boolean operators (e.g., `OR`), we were able to broaden our search to encompass a wide range of relevant literature while ensuring comprehensive coverage of the topics of interest. Human subjects approval was not necessary because there was no participant engagement.

### Eligibility criteria

Experimental, observational, and review articles published in- or representing individuals from Sub-Saharan Africa, the Caribbean and Latin America were considered in this review. All studies were included regardless of methodological approaches (e.g., quantitative, qualitative, mixed methods). All selected studies addressed both HIV stigma and additional forms of stigma; some studies explicitly referred to “intersectional stigma,” while others discussed supplementary stigmas in addition to HIV-related stigma (e.g., HIV stigma and sexual orientation stigma).

### Screening, data extraction and data items

We used CADIMA, an open-access software designed to support systematic reviews, scoping reviews and evidence synthesis. CADIMA facilitated our systematic review by streamlining literature screening, data extraction, and synthesis through a structured interface that allowed for dual independent reviews and standardized templates, minimizing bias and errors [37]. Its detailed logs and documentation capabilities ensured transparency and reproducibility, thereby enhancing the credibility and reliability of our study [37]. We uploaded the PICO/PECO criteria into CADIMA, which played a crucial role in ensuring that our screening, data extraction, and synthesis processes were aligned with our research objectives.

To minimize the impact of publication bias in our study, we implemented several strategies. First, we conducted an extensive search across multiple databases, including both peer-reviewed journals and gray literature sources such as conference proceedings, reports, and dissertations. This comprehensive approach helped us capture a broader range of studies, including those that may not have been published in traditional academic journals. Additionally, we actively sought feedback from the CAB, aiming to identify missing studies or relevant studies that may not have been published due to non-significant or null results. We also performed handsearching of key journals and conference proceedings and checked the reference lists of included studies to identify additional relevant studies that might have been missed in the database searches. By uploading our PICO/PECO criteria into CADIMA, we ensured a systematic and transparent screening process, with CADIMA’s detailed logs and documentation capabilities allowing us to track and report all included and excluded studies, providing a clear audit trail. Despite these efforts, we recognize that publication bias can never be entirely eliminated. However, by employing these strategies, we aimed to reduce its impact and enhance the robustness and credibility of our findings.

Two reviewers first screened the articles for duplication and inclusion/exclusion criteria. Team meetings were held to fine-tune the screening approach. Studies that passed the title and abstract screening were then assessed using full text to determine eligibility. An inter-rater reliability (IRR) [38] of 0.92 was reached by two reviewers (Authors NM and LMB) using Cohen’s Kappa after assessing 13% of the articles. After all the articles were reviewed, the study characteristics were extracted and compiled into a single spreadsheet for additional validation and coding. Extracted data focused on study characteristics (e.g., country, study design), population characteristics (e.g., gender, sexual identity), identified intersecting stigmas, and main findings/results. Simple descriptive statistics were calculated to summarize the characteristics of research and data.

## Results

### Overview

**Fig 1:**
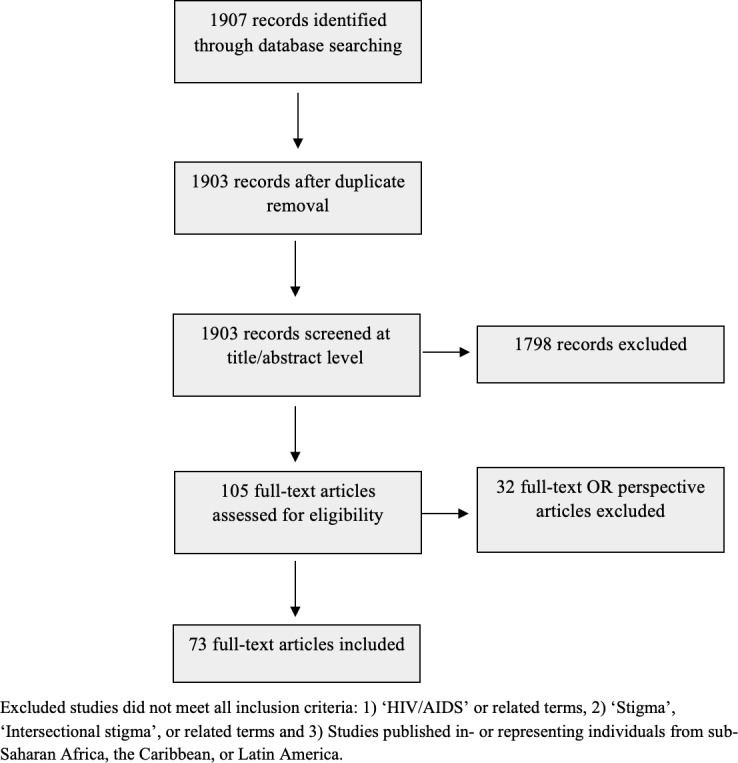
PRISMA Flowchart of study selection process. x-axis: none, y-axis: none.

Our search yielded 1,907 potentially relevant records. After implementing the inclusion criteria and removing duplicates, 105 articles were retained and assessed for eligibility. Of these, 32 articles were excluded after a full text review, leaving 73 studies that were included. The flow of articles in the selection process are presented in Fig 1.

### Study characteristics of included studies

**Fig 2:**
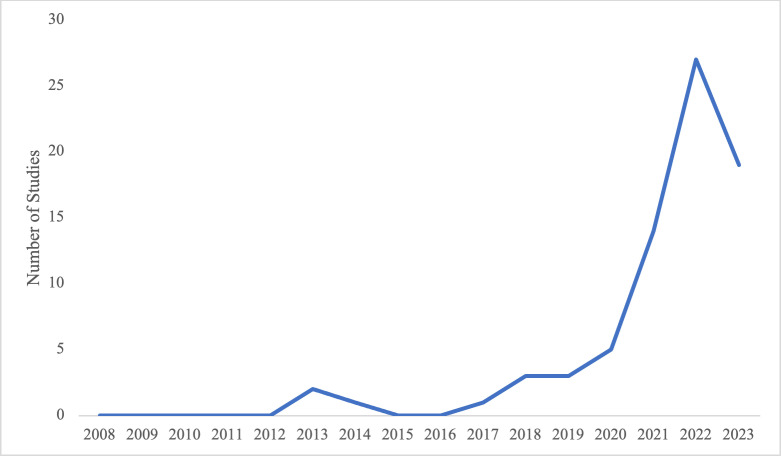
Number of Intersectional Stigma Studies by Year. X-axis: year, y-axis: number of studies.

The number of publications in sub-Saharan Africa, Latin America and the Caribbean on intersectional/intersecting HIV stigma has increased rapidly, from no publications in 2008 to 27 in 2022 (Fig 2). We identified 19 studies from January to April, 2023—which is less than half of the year, suggesting that the publication rate will be higher than in 2022. Approximately 16% were intervention studies and 84% were non-intervention studies. Of the intervention studies, 50% were effectiveness/preliminary effectiveness quantitative studies and 50% were implementation/formative intervention development research (qualitative and mixed methods) studies. Of the non-intervention studies, about half (49%) were exploratory (qualitative/mixed methods) studies, 28% were observational (quantitative) studies, and 23% were review articles.

Nearly three-fourths (73%) of identified studies were conducted in sub-Saharan Africa, 14% in Latin America, and 13% in the Caribbean. Studies that covered populations in two different regions were counted twice to ensure that the representation of research efforts and gaps was comprehensive and reflective of the actual distribution of studies across regions [6,7,39–42]. Among the studies in Sub-Saharan Africa, many were conducted in South Africa (24%) and Uganda (17%). In Latin America, many studies were conducted in Brazil (37%) and in the Caribbean, two-thirds of studies were conducted in the Dominican Republic (67%).

The populations of interest in these studies varied, with 59% focusing on people living with HIV (PLWH) including both adults and adolescents, and 41% on the general population without HIV. The latter group included sexual and gender minority (SGM) such as individuals who identify as lesbian, gay, bisexual, asexual, transgender, two-spirit, queer, and/or intersex, as well as adolescents and youth, healthcare and social service providers, and sex workers. Individuals often encountered compounded marginalization and social exclusion due to intersecting stigmas related to chronic diseases (e.g., cancer, skin disease), other infectious diseases (e.g., tuberculosis), socioeconomic status (e.g., poverty, homelessness), gender identity, sexual orientation, mental health, sex work, migrant/refugee status, pregnancy, incarceration, and other various demographic characteristics. Summary of study characteristics and intersecting stigmas are presented in Table 1.

**Table 1:** Summary characteristics of included studies.

Author	Intervention? Yes/No	Study Design	Region	Country	Population of Interest	Intersecting Stigmas
Tsang, 2019 [46]	No	Exploratory (Qualitative/Mixed Methods)	Sub-Saharan Africa	Zimbabwe	MSM	HIV Stigma Sexual Orientation
Ndirangu, 2022 [55]	Yes	Implementation/ Formative Research (Qualitative/Mixed Methods)	Sub-Saharan Africa	South Africa	Women with HIV	HIV Stigma Community Level Stigmas
Berner-Rodoreda, 2021 [49]	No	Exploratory (Qualitative/Mixed Methods)	Sub-Saharan Africa	Malawi	Men with HIV	HIV Stigma (Anticipated and Internalized Stigma)
Pepper, 2023 [58]	No	Exploratory (Qualitative/Mixed Methods)	Sub-Saharan Africa	South Africa	Women with HIV	HIV Stigma Tuberculosis Poverty
Magidson, 2022 [56]	No	Exploratory (Qualitative/Mixed Methods)	Sub-Saharan Africa	South Africa	PLWH Stakeholders from HIV and Substance Use Disorder Organizations	HIV Stigma Substance Use Disorder
Regenauer, 2020 [78]	No	Exploratory (Qualitative/Mixed Methods)	Sub-Saharan Africa	South Africa	PLWH and Problematic Substance Use	HIV Stigma Substance Use Disorder
Kennedy, 2013 [64]	No	Exploratory (Qualitative/Mixed Methods)	Sub-Saharan Africa	Swaziland/ Eswatini	MSM	HIV Stigma Sexual Orientation
Mujugira, 2021 [65]	No	Observational (Quantitative)	Sub-Saharan Africa	Uganda	Transgender Men	HIV Stigma Sexual Orientation Structural HIV Stigma
Yang, 2022 [48]	Yes	Effectiveness/ Preliminary Effectiveness (Quantitative)	Sub-Saharan Africa	Botswana	Pregnant women living with HIV	HIV Stigma Gender Pregnancy
Almeida, 2021 [68]	No	Observational (Quantitative)	Sub-Saharan Africa	Uganda	PLWH	HIV Stigma (Internalized, Anticipated and Enacted Stigma)
Logie, 2022 [9]	No	Review				HIV Stigma Race Sexual Orientation Substance Use Disorder Gender Homelessness Sex Work Socioeconomic Status
Stangl, 2013 [39]	No	Review	Sub-Saharan Africa Latin America Caribbean	Unspecified		HIV Stigma Structural
Atkins, 2022 [131]	No	Observational (Quantitative)	Sub-Saharan Africa	Kenya	MSM Female Sex Workers Adolescent girls and young women	PrEP-Related Stigma Sex Work
Budhwani, 2022 [89]	Yes	Effectiveness/ Preliminary Effectiveness (Quantitative)	Caribbean	Dominican Republic	MSM with HIV Transgender women with HIV Healthcare workers	HIV Stigma Sexual Orientation Gender Identity
Sharma, 2022 [67]	No	Exploratory (Qualitative/Mixed Methods)	Sub-Saharan Africa	Uganda	Youth with HIV	HIV Stigma Gender and Identity Stigma Age (Youth)
Ferraz, 2019 [83]	Yes	Implementation/ Formative Research (Qualitative/Mixed Methods)	Latin America	Brazil	General Population	HIV Stigma Sexual and Gender Identity
Khan, 2022 [66]	No	Exploratory (Qualitative/Mixed Methods)	Sub-Saharan Africa	Kenya	Youth Healthcare workers Stakeholders	HIV Stigma Age (Youth)
Viswasam, 2020 [40]	No	Review	Sub-Saharan Africa Latin America Caribbean	Unspecified		HIV Stigma (Enacted and Anticipated)
Mukamana, 2022 [117]	No	Exploratory (Qualitative/Mixed Methods)	Sub-Saharan Africa	Rwanda	Women with HIV	HIV Stigma (Enacted or Experienced) Community HIV Stigma (Anticipated & Internalized) Structural Stigma
Regenauer, 2022 [112]	No	Observational (Quantitative)	Sub-Saharan Africa	South Africa	PLWH	HIV Stigma Substance Use
Elafros, 2018 [80]	No	Observational (Quantitative)	Sub-Saharan Africa	Zambia	PLWH	HIV Stigma Epilepsy
Kimera, 2020 [69]	No	Exploratory (Qualitative/Mixed Methods)	Sub-Saharan Africa	Uganda	Youth with HIV	HIV Stigma Age (Youth)
dela Cruz, 2023 [70]	No	Review	Sub-Saharan Africa	Unspecified	Migrants form Sub-Saharan African Countries with HIV	HIV Stigma Race Migrant Status Gender
Rich, 2022 [63]	No	Exploratory (Qualitative/Mixed Methods)	Sub-Saharan Africa	Zimbabwe	Youth and Young adults with HIV	HIV Stigma Age (Youth)
Machingura, 2023 [93]	Yes	Implementation/ Formative Research (Qualitative/Mixed Methods)	Sub-Saharan Africa	Zimbabwe	Young Women	Poverty Sex Work
Cazeiro, 2023 [132]	No	Review				HIV Stigma Race Gender Class Sexual Orientation
Treves-Kagan, 2017 [133]	No	Observational (Quantitative)	Sub-Saharan Africa	South Africa	General Population	Individual HIV Stigma Community HIV Stigma
Meek, 2022 [54]	No	Exploratory (Qualitative/Mixed Methods)	Sub-Saharan Africa	Zambia	Adolescent girls and young women with HIV	HIV Stigma Age (Youth and Young adults)
Brown, 2021^4^	No	Review				HIV Stigma Age (Older adults)
Maiorana, 2023 [60]	No	Exploratory (Qualitative/Mixed Methods)	Latin America	Puerto Rico Mexico	Gay and Bisexual Men	HIV Stigma Sexual Orientation Limited Economic Resources Loss of Family Lack of Support No Housing
Abubakari, 2021 [97]	Yes	Effectiveness/ Preliminary Effectiveness (Quantitative)	Sub-Saharan Africa	Ghana	MSM	HIV Stigma Sexual Orientation
Ndagire, 2023 [110]	No	Exploratory (Qualitative/Mixed Methods)	Sub-Saharan Africa	Uganda	PLWH Provider	HIV Stigma Mental Health
Karver, 2022 [6]	No	Review	Sub-Saharan Africa Caribbean	Botswana Dominican Republic		HIV Stigma Gender Identity Race/ Ethnicity Sexual Orientation Substance Use Sex Work Sexually Transmitted Infection
Chimoyi, 2021 [134]	No	Exploratory (Qualitative/Mixed Methods)	Sub-Saharan Africa	South Africa	PLWH	Internalized HIV Stigma Community HIV Stigma
Skovdal, 2022 [135]	No	Exploratory (Qualitative/Mixed Methods)	Sub-Saharan Africa	Zimbabwe	Adolescent girls and Young women	Sexuality Stigma PrEP-Related Stigma
Pratt, 2023 [45]	Yes	Implementation/ Formative Research (Qualitative/Mixed Methods)	Sub-Saharan Africa	Uganda	Women with HIV	HIV Stigma Infertility-Related Stigma
Bergman, 2023 [81]	No	Review	Sub-Saharan Africa	Malawi Botswana South Africa Uganda Zambia Nigeria Kenya Tanzania Swaziland Cameroon Ghana	PLWH	HIV Stigma Gender Tuberculosis Age (Older Adults) Sexual Orientation Substance Use Disorder Socioeconomic Status Race
Hargreaves, 2018 [136]	No	Observational (Quantitative)	Sub-Saharan Africa	Zambia South Africa	PLWH	HIV Stigma (Internalized and Experienced)
Muñoz-Laboy, 2022 [71]	No	Observational (Quantitative)	Latin America	Puerto Rico	PLWH	HIV Stigma Race/ Ethnicity
Logie, 2021 [88]	No	Exploratory (Qualitative/Mixed Methods)	Sub-Saharan Africa	Uganda	Youth and Young adults	HIV Stigma Sex Work Age (Youth) Refugee Status Sexual Orientation Gender
Woznica, 2021 [47]	No	Exploratory (Qualitative/Mixed Methods)	Sub-Saharan Africa	South Africa	Previously Incarcerated individuals with HIV	HIV Stigma Incarceration Stigma
Daniel, 2021 [137]	No	Observational (Quantitative)	Caribbean	Haiti	Women with HIV	HIV Stigma Depression Sexual Relationship Power
Embleton, 2023 [44]	No	Exploratory (Qualitative/Mixed Methods)	Sub-Saharan Africa	Unspecified	Adolescents with HIV and without HIV	HIV Stigma Intimate Partner/Sexual Violence Refugee Status Gender Age Class-Related Orphaned Homelessness Sex Work
Abubakari, 2021 [97]	No	Review	Sub-Saharan Africa	Unspecified	MSM WSW	HIV Stigma Sexual Orientation
Lyons, 2020 [96]	Yes	Effectiveness/ Preliminary Effectiveness (Quantitative)	Sub-Saharan Africa	Senegal	Female Sex workers Cisgender MSM Transgender persons who have sex with men	HIV Stigma Healthcare Stigma
Mburu, 2014 [50]	No	Exploratory (Qualitative/Mixed Methods)	Sub-Saharan Africa	Uganda	PLWH	HIV Stigma Masculinity
Hsieh, 2022 [51]	No	Review	Latin America	Unspecified		HIV Stigma Age (Older Adults)
Brandelli Costa, 2022 [138]	No	Observational (Quantitative)	Latin America	Brazil	PLWH	HIV Stigma Healthcare Stigma
Kumwenda, 2023 [57]	No	Exploratory (Qualitative/Mixed Methods)	Sub-Saharan Africa	Malawi	PLWH Transgender person with disability Female sex worker MSM Healthcare workers/providers/social services Other Stakeholders (e.g., substance use disorder organizations, decision/policy makers)	HIV Stigma Poverty Gender Age Disability
Philip, 2023 [139]	No	Review	Sub-Saharan Africa	Ghana Uganda Zambia South Africa	PLWH	HIV Stigma Disability Gender Stigma Poverty
Mukerji, 2023 [41]	No	Review	Sub-Saharan Africa Caribbean	South Africa Uganda Malawi Kenya Dominican Republic Tanzania Swaziland Nigeria Zimbabwe Cameroon Ghana Ethiopia	Women with HIV	HIV Stigma Discrimination Domestic Violence
Nelson, 2021 [95]	Yes	Effectiveness/ Preliminary Effectiveness (Quantitative)	Sub-Saharan Africa	Ghana	MSM	HIV Stigma Sexual Orientation Healthcare Stigma
Saalim, 2023 [53]	No	Exploratory (Qualitative/Mixed Methods)	Sub-Saharan Africa	Ghana	MSM	HIV Stigma Sexual Orientation
da Silva Sousa, 2022 [72]	No	Exploratory (Qualitative/Mixed Methods)	Latin America	Brazil	PLWH	HIV Stigma Aging Sexual Orientation Disability
Yang, 2021 [140]	No	Exploratory (Qualitative/Mixed Methods)	Sub-Saharan Africa	Botswana	Women with HIV	HIV Stigma Gender
Ndione, 2022 [141]	No	Exploratory (Qualitative/Mixed Methods)	Sub-Saharan Africa	Senegal	MSM Healthcare Providers	HIV Stigma Sexual Orientation
Wadley, 2023 [142]	No	Observational (Quantitative)	Sub-Saharan Africa	South Africa	PLWH	HIV Stigma Chronic Pain
Knight, 2022 [52]	No	Exploratory (Qualitative/Mixed Methods)	Sub-Saharan Africa	South Africa	PLWH	HIV Stigma Age (Older Age)
Barrington, 2023 [143]	No	Observational (Quantitative)	Caribbean	Dominican Republic	Transwomen Sex Worker	HIV Stigma Sex Work Transgender
Jackson-Best, 2018 [7]	No	Review	Sub-Saharan Africa Latin America Caribbean	Unspecified		HIV Stigma Mental Health Disability Structural/Institutional Stigma
Goldenberg, 2021 [73]	No	Observational (Quantitative)	Caribbean	Dominican Republic	Transgender Women Sex Workers	HIV Stigma Sex Work Transgender
Meyer, 2023 [111]	No	Observational (Quantitative)	Sub-Saharan Africa	South Africa	PLWH and General Population	HIV Stigma Mental Health
Collier, 2022 [92]	Yes	Implementation/ Formative Research (Qualitative/Mixed Methods)	Sub-Saharan Africa	Kenya	PLWH	HIV Stigma Cancer Skin Disease
Hargreaves, 2022 [94]	Yes	Effectiveness/ Preliminary Effectiveness (Quantitative)	Sub-Saharan Africa	Zambia South Africa	PLWH	HIV Stigma Healthcare Stigma
MacLean, 2021 [74]	No	Review	Sub-Saharan Africa	South Africa	Mothers and Pregnant Women with HIV Adolescents	HIV Stigma Mental Health
Sileo, 2021 [82]	No	Observational (Quantitative)	Sub-Saharan Africa	Uganda	Men with HIV	HIV Stigma Gender
Johnson, 2019 [42]	No	Exploratory (Qualitative/Mixed Methods)	Latin America Caribbean	Unspecified	PLWH Older Gay/Bisexual Men Heterosexual men Heterosexual and Bisexual Women	HIV Stigma Sexual Orientation Age (Older Adults) Substance Use
Ulanja, 2019 [75]	No	Observational (Quantitative)	Sub-Saharan Africa	Cote d’Ivoire	MSM	HIV Stigma Healthcare Stigma
Munson, 2021 [84]	No	Exploratory (Qualitative/Mixed Methods)	Latin America	Guatemala	PLWH Gay and Bisexual Men Transgender Women	HIV Stigma Sexual Orientation Gender Identity
Nyblade, 2022 [76]	Yes	Implementation/ Formative Research (Qualitative/Mixed Methods)	Sub-Saharan Africa	Ghana	MSM Healthcare Providers	HIV Stigma Sexual Orientation Gender Identity
Stockton, 2023 [77]	No	Observational (Quantitative)	Sub-Saharan Africa	Kenya	Female Sex Workers	HIV Stigma Mental Health Sex Work
Bergam, 2022 [144]	No	Exploratory (Qualitative/Mixed Methods)	Sub-Saharan Africa	South Africa	Women	HIV Stigma Gender PrEP-Related Stigma
Diop, 2023 [113]	No	Exploratory (Qualitative/Mixed Methods)	Sub-Saharan Africa	Uganda	Youth	HIV Stigma Age (Youth) Migrant Status

### Drivers of stigma

Of the 73 studies, 18 identified the drivers of stigma. Stigma was propagated through Structural factors [4,43–54], institutional factors [46,51,53–57], and socio-demographic factors 57, 58]. The studies suggested that these drivers can interact with one another to create complex dynamics that reinforce stigma.

#### Structural factors.

Structural factors in this study refer to the broader, systemic elements that shape and influence societal conditions and individual behaviors [59]. These include economic systems, political frameworks, legal regulations, organizational policies, and other factors that influence the larger social and organizational frameworks such as culture, social support and peer support [59]. The collective beliefs, values, customs and behaviors of a group, referred to as culture, along with social conventions, which dictate acceptable behavior in a society or specific social setting, shape the standards of what is deemed socially and culturally acceptable or unacceptable [43,46,48]. For instance, some beliefs among sub-Saharan Africans include same-sex sexual behavior being perceived as “un-African, introduced by the Whites” and if a person has HIV their life is over [43,48]. These beliefs play a substantial role in influencing policies and perspectives on issues such as HIV, sexual orientation, gender identity and gender norms.

Both the prevalence of religion and the political orientation and values of the media in sub-Saharan Africa, Latin America, and the Caribbean play a pivotal role in molding social discourse [46,53]. In Ghana, Christianity and Islam view lifestyle practices associated with SGM as sinful and non-Ghanian [43]. This perspective has negatively impacted health seeking behaviors and utilization among SGM individuals [43]. Similarly, in Zimbabwe the media has reportedly been hostile to SGM male sex workers painting them as “abnormal and unclean” [46]. These media portrayals reflect underlying societal values and political orientations, contributing to the stigmatization and marginalization of SGM individuals.

National laws and religious institutions can reinforce and sustain damaging stereotypes that continue to negatively shape the social narrative, and impact resources that could help address HIV outcomes [46,53]. In Zimbabwe, male sex workers were excluded from national HIV prevention and treatment programs, leading to limited use of health care resources and increased vulnerabilities to HIV [46]. Other studies reveal that negative perceptions of SGM such as blaming SGM individuals for spreading HIV in Ghanaian communities or being negatively labeled, drive continued stigmatization of sexual minority men and male sex workers [46,53]. In Zimbabwe, legal structural systems prevent male sex workers from seeking healthcare out of fear of being arrested because being a SGM is a crime [46].

### Structural moderators of stigma

Social and peer support can be significant factors in moderating the effects of stigma. Unlike other structural factors that primarily describe conditions or contexts, social and peer support actively influence the impact of stigma on individuals. For example, in Uganda, participating in peer support groups related to HIV can inadvertently isolate individuals with the condition, amplifying the discrimination they face because they are identifiable when they meet together [50]. On the other hand, social support can mitigate the effects of stigma, as shown in a study in Botswana among pregnant women living with HIV [48]. Moreover, in Mexico, sexual minority individuals who disclosed their HIV status to family and friends were able to obtain support to cope with stigma [60]. In South Africa, women more so than men, have reported a notable absence of support from family and friends [52]. This lack of support was linked to feelings of rejection, heightened intersectional stigma, and overall poorer health outcomes [52]. Similar findings regarding social support has also been documented in studies conducted in Latin America as well [51].

#### Institutional factors.

Institutional factors – structural and organizational elements within institutions that perpetuate stigma – are reported to further perpetuate stigma and impact those seeking HIV services [46,54]. For instance, healthcare workers in Zambia report that intersectional stigma in the clinics is driven by the structural environments of the facility [54]. Clinics are designed with limited privacy, leading to breaches in confidentiality and deterring people from being forthcoming about their health prognosis or visiting the clinic [54,55]. One study highlighted environmental stigmas in a clinic’s practice of using differently colored medical records and segregating HIV patients in hospital sections, inadvertently leading to disclosures and heightened stigmatization [55]. This can be problematic, as clinic infrastructure can exacerbate the stigmas already experienced by PLWH, particularly concerning privacy concerns. This is especially pertinent when individuals are concealing not only their HIV status but also other identities, such as SGM. Furthermore, it is worth noting that these studies [54,55] were conducted in public clinics serving low-income individuals, which may be associated with the stigmatizing infrastructure of these clinics.

Additional factors within healthcare that worsen challenges for individuals living with HIV include insufficient integration of HIV care into health facilities for those who also use substances [56]. Other studies point that issues such as, social preferentialism based on economic status, gender, age (with a particular focus on the challenges faced by the elderly in HIV care) and other social stratifies within the healthcare system, were identified as factors driving stigma [51,57].

Research from South Africa assessing the intersection of substance use disorders and HIV, suggests that healthcare structures that incorporate peer-based care have the potential to transform the culture to be more inclusive and less stigmatizing [56]. These structures can provide patients with guidance on advocating for themselves during HIV visits, particularly in managing anticipated stigma from healthcare workers [56].

#### Socio-demographic factors.

Socioeconomic factors – defined as the characteristics, of individuals or populations, such as age, gender, income, education, and ethnicity, that influence stigma – are an important facet of intersectional HIV stigma. Those in poverty struggle meeting their basic needs such as food and housing, and often have limited access to education and healthcare resources, which are critical for accurate information dissemination about HIV [57,58]. This lack of access often results in misinformation and misconceptions about HIV, contributing to stigmatization of others and self-stigma [61–63]. Furthermore, those living in poverty often face limited access to proper healthcare services, including HIV testing, treatment, and prevention [57,58]. This lack of resources perpetuates the cycle of stigma by reinforcing the belief that HIV is predominantly a problem for those in disadvantaged socioeconomic positions. In addition, there is a lack of consideration for individuals facing financial constraints impacting antiretroviral therapy (ART) adherence [55]. Study participants note that feeling financially unstable restricts their capacity to address additional concerns, such as taking medications or having the financial resources for transportation [51,55,56]. This intersectional stigma not only affects how society perceives individuals with HIV but also impacts on how those individuals perceive themselves, further exacerbating the challenges they face in seeking support and care.

Gender-based stigma can lead to violence and abuse, ranging from intimate partner violence [41,55,58], physical violence [40,53,64] to transphobic rape [65]. Women and gender minority individuals often have increased physical and verbal abuse, as well as emotional and psychological abuse to exert control and instill fear [40,41,53,64]. Transgender individuals face an elevated risk of sexual violence, including transphobic rape and hate crimes driven by societal stigma and discrimination [65]. This stigma can lead to higher incidences of rape and sexual assault, as perpetrators believe that individuals are less likely to report these crimes due to fear of further stigmatization or a lack of protection from law enforcement even if the crimes are reported [64].

Young men often find themselves pressured to conform to narrow masculine norms, further contributing to the cycle of stigmatization [49], and leading to lower levels of HIV-related healthcare and support-seeking [49]. Societal expectations often dictate that men should embody strength, invulnerability, and sexual prowess, creating an environment where acknowledging vulnerabilities, seeking help, or discussing sensitive health issues like HIV can be perceived as a sign of weakness or a deviation from traditional masculinity. This pressure to conform to rigid gender roles not only hinders open discussion about HIV but also prevents men from accessing necessary physical and mental health care, perpetuating the intersectional stigma associated with HIV.

### Impact of stigma

Intersectional HIV stigma has a multifaceted impact on individuals and communities across regions. It obstructs access to vital healthcare services and information [57,64,66,67], engendering avoidance of healthcare/health facilities [54,55,57,66], poor care retention [55], and limited peer support participation [56,57,64,65]. This, in turn, diminishes the quality of health and general services available to those affected by or at risk for HIV.

The rejection of SGM within communities can pose significant obstacles for individuals in accessing suitable healthcare, HIV prevention measures, and support systems [45,46,48,49]. Such cultural beliefs can also lead to low healthcare utilization and HIV testing and lack of disclosure of HIV status or sexual orientation [43,50]. Cultural and social norms also have implications for socioeconomic stability, including restricting job opportunities—which in turn can detrimentally affect treatment adherence, as maintaining one’s health often requires financial resources (e.g., for transport to health care facilities) [44,47].

One of the most consequential impacts of intersectional stigma in the scoping review was around mental health, including depressive symptoms, loneliness, and loss of self-esteem [40,51,54,57,63–65,67–77]. The stigma associated with both HIV and mental health not only prompts but also intensifies behaviors that may contribute to HIV transmission and poor health maintenance for those with HIV. This includes a rise in substance use and engaging in unprotected sex, when compared to situations where only one of the issues – either mental health or HIV – is stigmatized [40,65,68,77–79]. The subsequent effect of intersectional stigmas on individuals’ physical health, affects antiretroviral adherence, rendering individuals more susceptible to comorbid diseases and increasing hospitalization rates [58,63,68,72,80–82]. For example, in South Africa, individuals have reported experiencing mistreatment from HIV clinic staff while collecting their medication due to issues related to their alcohol and other drug use [78]. Consequently, this has discouraged them from seeking further care [78].

Intersectional stigma (intersecting HIV stigma with age, gender, mental health, incarceration status) also permeates social realms, suppresses disclosure, and diminishes social support and resilience, with individuals often encountering discrimination in all aspects of life [9,39,47,50,65,67,70,83]. Intersectional stigma (intersecting HIV stigma with poverty, age, gender, SGM) has been reported to also impede educational pursuits, limit economic opportunities, and hamper the ability to fulfill expected social roles [50,57,69,81,84]. For example, in Brazil, individuals who identify as SGM and also have HIV are apprehensive about revealing both aspects of their status out of fear of potential job loss, difficulties securing employment or facing rejection in educational settings [72].

### Impact of region

Place is an important and understudied analytical unit in HIV prevention research on intersectional stigma. Diverse regions and environments exhibit distinct legal frameworks, levels of violence, economic disparities, social instability, joblessness, limited resources, and varying degrees of community unity [85,86]. These elements collectively contribute to an increased susceptibility to HIV for specific regions and subgroups of individuals within them [85,86]. In counties like Uganda, for instance, criminalization of SGM individuals can impede receipt of HIV treatment (e.g., individuals are reluctant to seek care) or preventative methods for HIV (such as pre-exposure prophylaxis (PrEP)) [87]. Moreover, HIV stigma that is intertwined with ‘place’ and its contributing factors such as policies and resources, too often create intersectional stigma that impacts individuals’ experiences. For example, in areas of high levels of violence (e.g., against SGM individuals or sex workers), or social instability, accessing HIV prevention and treatment services might be challenging for some individuals due to safety concerns or disrupted healthcare systems [41,55,88]. In South Africa for instance, women who live in economically disadvantaged settings faced the fear of robbery or harm while seeking their ART medication, leading to a reluctance to go to clinics [55].

Economic disparities and joblessness can also exacerbate vulnerability to HIV, as individuals in these environments may engage in behaviors that increase HIV risk due to limited opportunities, such as transactional sex for survival, further amplifying the intersectional stigma associated with both poverty and HIV [47,65]. Moreover, policies and resources allocated to specific places can inadvertently perpetuate stigma by either supporting or neglecting certain communities, thereby influencing the perception and treatment of individuals living with HIV within those locations [46].

#### Latin America and the Caribbean.

Of the studies conducted in Latin America and the Caribbean, 33% were on SGM individuals, and 17% sex workers, while the rest were on the general population of people with HIV (42%) and women (8%). Of the studies in Latin America and the Caribbean that specified the country in which the study was conducted, the majority in the Caribbean were conducted in the Dominican Republic (83%) while those in Latin America were predominately in Brazil (50%) and Puerto Rico (33%).

Research indicates a disproportionate burden of intersectional stigma, including violence, among sex workers and SGM communities across regions. In Guatemala, the intersecting stigmas of gender identity and sex work significantly amplify the challenges faced by sexual minority male and transgender sex workers. Compared to sexually minority men not involved in sex work, those engaged in sex work had more than a sixfold increased likelihood of experiencing forced sex, physical abuse and discrimination [40]. This underscores the compounded impact of intersectional stigma, contributing to elevated risks of both violence and HIV within this demographic. In the Dominican Republic, policies that perpetuate stigma towards those with HIV and those who are SGM individuals are codified into laws [89]. These laws have been internalized by healthcare workers and reflected toward patients in discriminatory practices thus showing the impact of laws into the healthcare system [89]. Ongoing studies are aiming to increase understanding of HIV-related and intersectional stigmas by bringing together healthcare workers and PLWH [89]. In Brazil, despite transphobia being recognized as a crime since 2019, the country continues to have the highest number of murders targeting transgender and queer individuals globally [90]. This legislation in Brazil, coupled with high community-level HIV stigma, has led to significant challenges for those who are SGM and have HIV [72]. For instance, the societal taboo associated with sexuality in old age poses a socio-cultural challenge, influencing social interactions and exacerbating discrimination against individuals doubly stigmatized for being both older and a SGM [72]. This makes it difficult to effectively address HIV among older people.

#### Sub-Saharan Africa.

Of the studies conducted in Sub-Saharan Africa, the majority focused on PLWH (58%), followed by the SGM community (24%), sex workers (8%), the general population (20%), and previously incarcerated individuals (2%). (Some studies focused on more than one population and were counted twice.)

African nations have the highest number of anti-SGM laws globally, perpetuating a climate of stigma and discrimination [53,91]. Furthermore, some nations in the region criminalize sex work, compounding the challenges faced by marginalized communities [40,46]. These policies directly impact the overall health and well-being of numerous individuals especially those with HIV. For instance, in South Africa, sex work criminalization leads to a convergence of issues including homelessness, violence and substance abuse [40]. In Zimbabwe, male sex workers are excluded from national HIV prevention and treatment programs, lacking dedicated funding and specific initiatives tailored to their needs and thus relied solely on information and resources provided by SGM-focused organizations [46]. Additionally, some religious institutions, and deeply ingrained cultural and gender norms further contribute to an environment that exacerbates discrimination and stigma [43,45].

### Interventions

The majority of the intervention studies were conducted between 2020 and 2023 [45,48,55,76,89,92–97]. Only one was conducted prior in 2019 [83] and two were protocol papers for ongoing studies [89,95]. Of these intervention studies, 33% focused on MSM, 25% on women with HIV, 16% on PLWH, 16% on the general population and 8% on female sex workers. Empirical studies revealed that targeted trainings or stigma mitigation interventions can decrease intersectional stigma [48,76,96,97]. These trainings included those at risk for and living with HIV and targeted the intersecting stigmas in relation to pregnancy and SGM identification. ART adherence and post-exposure prophylaxis (PEP) participation is influenced by intersectional stigmas [55,83]. Other studies identified varying factors, such as poverty, that can hinder programs designed to address intersecting stigma [45,93].

### Global CAB perspective

The primary point emphasized by the Global CAB, based on members’ experiences in their regions, own research [98–104], discussions with the local CAB, and review of the above review results, was that stigma manifests differently across countries and regions. The CAB noted that most intersectional stigma research has focused on key populations, such as SGM individuals, and identified a need to study populations affected by stigma at the intersection of socio-demographic characteristics related to age, gender, socio-economic status, and occupation. The CAB also suggested more attention to other sub-populations that experience stigma, including people with disabilities, people with mental health issues, and people with other health conditions (e.g., tuberculosis).

The CAB expressed that gender is an overarching, cross-cutting category, and that some intersectional stigmas may be based in religious and cultural norms related to traditional gender roles (e.g., about the inappropriateness of sex and sexuality for some groups). As discussed by CAB members from Botswana, Uganda, and Zambia, older women and youth, especially young adolescent girls (below the age of 18), as well as pregnant women, are assumed not be to sexually active based on cultural and religious norms and are stigmatized as promiscuous if they are. This means that national campaigns around HIV prevention may not be tailored for or be inclusive of these groups; for example, members of the CAB from Zambia and Botswana noted that PrEP has been marketed for sex workers and men who have sex with men, but in countries with generalized epidemics, everyone is at risk and can benefit from PrEP. Moreover, health care workers may not offer HIV prevention services (e.g., HIV testing and PrEP) or may negatively judge and question such individuals if they do request services. CAB members also discussed the related issue of gender-based violence, particularly in regions like Botswana, where high prevalence persists despite high resource allocation (e.g., freely available antiretroviral therapy) and protective policies around SGM individuals [105,106].

Another population affected by gender and age stigmas, especially in southern Africa, are older men of higher socio-economic status, who may have multiple partners in the context of generalized epidemics, but they may fear a loss of masculinity and status if they are diagnosed with HIV; thus, they may avoid HIV prevention and treatment services [101].

The CAB also mentioned the need to study intersectional stigma among mobile populations, which include occupations such as people involved in fishing, mining, cross-border trading, and immigrants. Mobile populations may be stigmatized because their communities are known to have high levels of HIV as well as poverty [107,108]. Because of their mobility, they also have low access to health care in general as well as HIV prevention services, as they travel far distances in rural areas, away from health care facilities, for their livelihood [107–109].

## Discussion

Our scoping review revealed that intersectional stigma studies in sub-Saharan Africa, Latin America, and the Caribbean have increased from 2008 to 2023. Of the 73 identified studies published, most were conducted in sub-Saharan Africa. The lower proportion of studies from Latin America and the Caribbean could be due to the estimated low prevalence of HIV in both regions [0.5% in Latin America and 1.2% in the Caribbean] compared to sub Saharan Africa [Western and Central Africa (1.1%) and Eastern and Southern Africa (5.9%)] [21].

The most commonly studied impact of intersectional stigma was on mental health and well-being. Mental health is a critical factor among PLWH because the psychological burden of managing a chronic illness, coupled with the stigma and discrimination associated with HIV, significantly impacting their overall well-being and quality of life [7,74,77,110,111]. In addition, intersectional stigma prompted behaviors that put people at risk for HIV, heightened incidence of violence and abuse, lowered rates of HIV disclosure, and restricted economic prospects. Studies reported that disclosure of HIV often increased intimate partner violence especially for women [41,44]. The compounded effects of stigma related to HIV, and substance use also led to enacted and anticipated stigma from care providers which acted as deterrent for people wanting to seek care and impacted viral load suppression [9,56,78,112].

In addition, studies in this review spanned various demographics, finding, consistent with research in other regions of the world, many focused on key populations (e.g., sex workers, SGM, people in prison) with higher incidence and prevalence rates of HIV [21]. However, other intersectional stigmas emerged that have received less attention in the overall literature, including chronic diseases (e.g., cancer, skin disease) [92], age(the youth and the elderly) [4,42,51,52,54,63,66,67,69,81,88,113], and alcohol and other drug use [6,9,42,56,57,78,81,112].

### Gaps in literature

This review identified several gaps in the literature. First, the articles in this review almost exclusively focused on intersectional stigma from the target’s perspective, not the enactors. While an intersectional perspective aids in understanding the experiences and consequences of living with multiple stigmatized identities, not understanding the enactors leaves a critical piece of the puzzle unexamined. Social psychology studies have shown that understanding the motivations, attitudes, and behaviors of the enactors of stigma is crucial for developing effective interventions and strategies to challenge and mitigate such discrimination [114–116]. Without this insight, efforts to address intersectional stigma may miss opportunities for meaningful change. Additionally, neglecting the perspective of enactors may inadvertently perpetuate and/or normalize stereotypes and misconceptions. While it is important to acknowledge that stigma can exist at multiple levels – including interpersonal, community and structural levels – this review highlights the need for a comprehensive examination that encompasses both the target and enactors perspectives. Multi-level stigma manifests in various ways such as through societal norms, institutional practices, and policy frameworks, which collectively drive and perpetuate discrimination. Therefore, we recommend that future global health research on intersectional stigma incorporates a holistic approach that examines these multiple levels and includes both target and enactors perspectives. More intervention studies need to be conducted to address stigma from its source, whether it be at the individual, community or structural level.

Secondly, while certain articles in this review acknowledged the presence of intersectional structural stigma [46,55,117], a notable portion did not, indicating a knowledge gap in the pursuit of equity. Emphasizing structural stigma, defined as the “societal-level conditions, cultural norms, laws, and institutional practices that constrain the opportunities, resources, and wellbeing for stigmatized populations,” underscores the importance of integrating ‘place’ as a metric [118]. Examining the spatial manifestation of intersectional stigma provides novel perspectives to the behavioral dimension of stigma. Place is conceptualized as a geographic area (e.g., region of the world, neighborhood) that shapes and is constructed by the lived experiences, practices, interactions and identities of those who inhabit and navigate a space [85,119]. This approach opens up innovative avenues for interventions aimed at rectifying disparities and curbing the perpetuation of stigma within policies, systems, social frameworks and institutions [85,120,121]. As an example, placing emphasis on structural stigma through the integration of ‘place’ as a metric can facilitate the recognition of societal-level conditions, cultural norms, laws, and institutional practices affecting stigmatized populations. Intervention studies can additionally focus on mitigating the persistence of stigma within policies, systems, and social frameworks.

Lastly, the studies in this review concentrated solely on the negative impacts of stigma, neglecting to explore the strengths, resistance, and empowerment that are also integral components of intersectionality [8]. Taking a deficit approach to HIV intersectional stigma may lead to a lack of recognition of the valuable knowledge, collective resources and skills within various communities and populations [122]. Therefore, we recommend that future studies place an emphasis on harnessing innate strengths of cultures, health care systems, and individuals to create opportunities at macro (advocacy), meso (social support and solidarity) and micro (resilience levels) [122–124].

### Global CAB recommendations: The way forward

The Global CAB made the following recommendations based on the scoping review and identification of key gaps in the research literature:

*Conduct social marketing research to (1) determine how to promote HIV services in the general population, across gender, age, and socio-demographic subgroups; and (2) to develop strategies that can be conveyed to local and national media about how to change the narrative about HIV from stigmatizing to compassionate*. CAB members from sub-Saharan Africa suggested developing positively framed messages that address HIV-related misconceptions that lead to stigma (e.g., HIV can affect anyone, everyone should know about PrEP, people with HIV should not be blamed) [125]. In Latin America and the Caribbean, CAB members suggested educating media outlets, that write sensationalizing and stigmatizing stories about people with HIV who are SGM, about how to be discuss HIV and SGM issues in a compassionate light [126]. Qualitative research is also needed to understand and design messages that resonate with general as well as key populations, to be inclusive of all groups across intersectionalities [127,128]. This recommendation aligns with the review’s findings that understanding the motivations and behaviors of not only the enactors of stigma, but the general population as well, is crucial for developing effective interventions. By focusing on how to convey positively framed messages that address HIV-related misconceptions, this recommendation directly aims to challenge and mitigate discrimination at its source.

#### Investigate the impact of anti-SGM+ laws on participation in HIV services and research.

The CAB expressed that punitive legislation [129,130] that criminalizes SGM individuals and people with HIV, such as in CAB member countries Uganda and Zambia, reinforces stigma and discrimination and will cause people to avoid HIV services and not engage with care. Ideas for data collection included documenting how people with HIV in Uganda from key populations have changed health care behaviors in response to such laws (e.g., avoidance of health care, which can lead to unsuppressed viral load and greater HIV incidence); conducting an online survey of researchers and health care providers to understand how such laws are impacting research and health care services; and meeting with institutional review board directors to determine best practices for safeguarding human rights in these contexts. Armed with empirical data on how the laws are affecting the country’s population (and the health of its population), researchers and community activists can be better advocates for changing laws within countries and globally. Moreover, such information can lead to intervention development, such as peer support and continuing medical education (about client-centered care that is accepting and respectful of all groups) that reduce stigma in the context of such laws. This recommendation is particularly pertinent given the review’s identification of a knowledge gap in understanding structural stigma. By document how punitive legislation affects health care behaviors and conducting surveys with researchers and health care providers, the CAB’s recommendation aims to provide empirical data that can be used to advocate for legal and policy changes.

#### Conduct research from the perspective of people affected by intersectional stigmas.

The CAB discussed the need to do in-depth qualitative research with people affected by intersectional stigma in order to answer a number of research questions, including: What are the contexts and mechanisms by which stigma affects health? How does internalized HIV stigma change and evolve over time, from initial denial to acceptance? What lessons can we learn for how people persevere in the face of extreme stigma and discrimination (e.g., Rwandan genocide, South African apartheid)? What are the barriers to HIV research participation among populations affected by intersectional stigma, and what safeguards must be put in place by institutional review boards? (For example, on this last point, a CAB member from Peru noted that transgender women may be harassed on public transportation when traveling to a study site and thus, institutional review boards should specify that transgender women be given funds for private transportation to research sites.) Such research could provide a firm basis for interventions, marketing, and policies to reduce intersectional stigma. This recommendation addresses the review’s results that current literature focuses almost exclusively on the targets’ perspective, neglecting the enactors. By conducting in-depth qualitative research to understand the contexts and mechanisms by which stigma affects health, this recommendation aims to provide a more comprehensive understanding that includes both the experiences of those stigmatized and the behaviors of those who enact stigma.

### Strengths and limitations

Our systematic literature search and review approach, combined with community engagement to interpret results, is a strength. Another strength is the focus on specific regions in the world to better understand the landscape of intersectional stigma. This study, however, was limited only to published studies written in English. Also, the limited number of published works in the different regions of interest (sub-Saharan Africa and Latin America and the Caribbean) made it difficult to draw region-specific conclusions.

## Conclusion

The results of this scoping review help to elucidate the landscape of intersectional stigma research in sub-Saharan Africa, Latin American and the Caribbean. This understanding is pivotal in determining the precise interventions and policies required to address the unique challenges encountered in these regions. Such a focused approach can pave the way for more impactful strategies, ultimately reducing stigma, enhancing healthcare accessibility, and improving the overall well-being of individuals navigating intersecting stigmatized identities in these areas.

## Supporting information

S1 Text Preferred Reporting Items for Systematic reviews and Meta-Analyses extension for Scoping Reviews (PRISMA-ScR) Checklist. x-axis: none, y-axis: none.
(DOCX)
